# Machine learning approach for pooled DNA sample calibration

**DOI:** 10.1186/s12859-015-0593-1

**Published:** 2015-07-09

**Authors:** Andrew D Hellicar, Ashfaqur Rahman, Daniel V Smith, John M Henshall

**Affiliations:** 1 CSIRO Computational Informatics, Castray Esplanade, Hobart, Australia; 2 CSIRO Agriculture Flagship, Armidale, Australia

**Keywords:** DNA pooling, Calibration, Machine learning, SNP

## Abstract

**Background:**

Despite ongoing reduction in genotyping costs, genomic studies involving large numbers of species with low economic value (such as Black Tiger prawns) remain cost prohibitive. In this scenario DNA pooling is an attractive option to reduce genotyping costs. However, genotyping of pooled samples comprising DNA from many individuals is challenging due to the presence of errors that exceed the allele frequency quantisation size and therefore cannot be simply corrected by clustering techniques. The solution to the calibration problem is a correction to the allele frequency to mitigate errors incurred in the measurement process. We highlight the limitations of the existing calibration solutions such as the fact they impose assumptions on the variation between allele frequencies 0, 0.5, and 1.0, and address a limited set of error types. We propose a novel machine learning method to address the limitations identified.

**Results:**

The approach is tested on SNPs genotyped with the Sequenom iPLEX platform and compared to existing state of the art calibration methods. The new method is capable of reducing the mean square error in allele frequency to half that achievable with existing approaches. Furthermore for the first time we demonstrate the importance of carefully considering the choice of training data when using calibration approaches built from pooled data.

**Conclusion:**

This paper demonstrates that improvements in pooled allele frequency estimates result if the genotyping platform is characterised at allele frequencies other than the homozygous and heterozygous cases. Techniques capable of incorporating such information are described along with aspects of implementation.

## Background

Recently the Illumina HiSeq X Ten [1] achieved a new low in per genome sequencing cost, continuing the ongoing reduction in cost per genome since 2001 [2]. These cost reductions now make it practical to genotype individuals in large association studies of humans. However, this is not the case for studies involving large populations of low economic value species where contemporary genotyping technology is cost prohibitive. The cost benefits achieved in [1] have not been realised on platforms based on alternative technology, such as Sequenom, and therefore pooling is still required in this scenario. This is evidenced by the ongoing use of DNA pooling in studies on low economic value species, specifically to reduce cost [3,4]. DNA pooling has been shown to provide a cost benefit over individual genotyping [5] and allows access to a broader community to enable genetic association studies.

Pooling techniques date back to 1943 when blood from soldiers was pooled for testing of disease [6] and pooling of DNA was first proposed in 1985 [7]. The field advanced rapidly and in 2002 a broad review of the approach (applied to SNP data) was published [8]. DNA pooling combines DNA from multiple individuals into a single sample which can be genotyped once, as opposed to genotyping each individual. This reduces the cost of genotyping by a factor equal to the number of individuals in the pooled sample. In general pooling strategies are more complex and involve the multiple genotyping of duplicate pools, the effiiency of pooling approaches is given in [8]. The general pooling approach changes the measurement from detecting whether or not a substance is present, to measuring the concentration of the substance. In the case of DNA pooling, the ‘substances’ are the discrete SNP genotypes *A* *A*,*A* *B*,*B* *B* with corresponding A-allele frequencies 1,1/2,0 and the ‘concentration’ is equivalent to the real valued A-allele frequency within the range [0, 1].

The most significant drawback of the pooling approach is the error incurred in the process of measuring the pool’s allele frequency. The impact of this error is illustrated in the context of a bi-allelic quantitative trait linkage study.

Given a population and a single trait of interest, two sub-populations (*α* and *β*) are identified exhibiting opposing extremes of the trait. From each sub-population a sub-set of individuals are selected, DNA acquired from each individual and combined in a single pooled sample representative of the respective subset. The two pooled samples are genotyped and their allele frequencies are compared. Both fixed and variable errors in the allele frequency measurement impact the power of such a study:

(1) $$ Z^{2}=\frac{\left(\,f_{\alpha}-f_{\beta} \right)^{2}}{(V_{\alpha}+V_{\beta}) }  $$

where Z is the study test statistic, *f*_*α*_ and *f*_*β*_ are the best estimates of the A-allele frequency of the two sub-populations, and *V*_*α*_ and *V*_*β*_ are the variances in *f*_*α*_ and *f*_*β*_. If the genotyping hardware response for a sample’s allele A and allele B are *H*_*A*_ and *H*_*B*_ respectively then typically the sample’s allele frequency (*f*) is calculated from:

(2) $$  f=\frac{H_{A}}{H_{A}+H_{B}}.  $$

Three main factors contribute to the allele frequency variation including: sampling error *E*_*s*_ (due to the limited pool size), sample construction error: *E*_*p*_ (due to non ideal pool constructing resulting from the unequal contributions of individuals to the pool sample) and allele frequency measurement error: *E*_*m*_ (due to chemistry and detection errors in the genotyping process). If the true sub-population allele frequency is *p*, then these errors result in a measured allele frequency *f*=*p*+*E*_*s*_+*E*_*p*_+*E*_*m*_. The variance introduced in *f* by approximating sub-population with *N* individuals is the expectation of the square error: $\overline {{E_{s}^{2}}}=p(1-p)/2N$. Similarly the unequal contributions to a pool from individual samples contribute to a variance component $\overline {{E_{p}^{2}}}=\tau p(1-p)/2N$ [9] where *τ* is the standard deviation in the fractions of the pool contributed by the individuals. A thorough analyses of these errors under different sampling conditions is given in [10]. Both these variance contributions can be reduced by increasing the pool size. Measurement error; however, is independent of pool size. Reducing measurement error requires averaging over multiple measurements, which reduces cost effectiveness of the pooling strategy. To resolve this issue, a range of calibration techniques have been proposed for *E*_*m*_ reduction. Three example approaches are k-correction [11], linear interpolation [12] and the polynomial-based probe specific correction (PPC) method [13].

Despite the fact that these methods were developed for different platforms, they all contain a number of similarities which allow them to be applied to data generated by the Sequenom platform. All existing calibration techniques have a mapping which takes as input the raw allele frequency resulting from the platform’s response to each of the two alleles present for a SNP. The Sequenom data is also available in this format. Furthermore the SNP specific corrections are based on the platform’s allele responses to multiple individuals for the SNP being corrected. Sequenom data can also be generated by multiple individuals to provide such a data set. To explain these techniques the following notation is adopted:

Given a SNP requiring calibration, and a set of AA homozygous individuals in the SNP, define $\overline {AA}=(\overline {H_{A}(AA)},\overline {H_{B}(AA)}$) where $\overline {H_{A}(AA)}$ and $\overline {H_{B}(AA)}$ are the average value for *H*_*A*_ and *H*_*B*_ over the AA homozygous set of individuals. Similarly $\overline {AB}$ and $\overline {BB}$ are average values defined for heterozygous *AB* and homozygous *BB* sets of individuals respectively. The measured allele frequency *f*, corresponding to points $\overline {AA}$, $\overline {AB}$, and $\overline {BB}$, are *f*_*AA*_, *f*_*AB*_, and *f*_*BB*_ respectively. The calibration techniques all map *f*_*AA*_ and *f*_*BB*_ into A-allele frequencies 1 and 0 respectively with calibration specific approaches between these values to map *f*_*AB*_ into A-allele frequency 0.5. How they achieve this varies between the methods.

k-correction was introduced to correct for error in the PCR process [11], specifically SNP dependent unequal amplification of alleles during PCR. The correction involves using $\overline {AB}$ to calculate ratio $k=\overline {H_{A}(AB)}/\overline {H_{B}(AB)}$, ideally *k*=1 in the absence of differential amplification. The ratio *k* is used to correct the distorted post-PCR measured quantities resulting in the following expression for calibrated allele frequency *f* ^′^:

(3) $$  f'=\frac{H_{A}}{H_{A}+kH_{B}}.  $$

k-correction approach can be applied to the Sequenom data without modification.

The piece-wise linear calibration approach of Illumina [12] involves four linear transformations of (*H*_*A*_,*H*_*B*_) corresponding to rotation, translation, shear and scale transformations. These transform $\overline {AA}$ and $\overline {BB}$ onto *H*_*A*_ and *H*_*B*_ axes respectively, with approximately equal amplitude. Finally a piecewise linear function maps angles on the (*H*_*A*_,*H*_*B*_) plane at points $\overline {AA}$, $\overline {AB}$, and $\overline {BB}$ onto A-allele frequencies 1, 0.5, and 0 respectively. The function linearly interpolates angles between these points, therefore the entire calibration process for pools involves a combination of the four transformations, calculating angle *a* *t* *a* *n*(*H*_*A*_/*H*_*B*_), and piece-wise linear interpolation. Our implementation of the piece-wise linear approach is similar; however to ensure consistency across the calibration methods we utilise the form given in Eq. 2 and the corrected allele frequency *f* ^′^ is:

(4) $$\begin{array}{*{20}l} &f'=\frac{f-f_{AB} }{ f_{AA} - f_{AB}} + \frac{1}{2} \frac{f_{AB} - f}{f_{AA}-f_{AB}} & f>f_{AB}\\ &f'=\frac{f-f_{BB} }{ f_{AB} - f_{BB}} & f<=f_{AB} \end{array} $$

Minor changes include the fact that the ratio in Equation (2) is used in calculating allele frequency, as opposed to the normalised angle (2/*p* *i*)*a* *t* *a* *n*(*H*_*A*_/*H*_*B*_). Dividing by *H*_*A*_ + *H*_*B*_ in Eq. 4 introduces a normalising factor, and enforcing the homozygous values to 0, 1 and heterozygous cluster centre to 0.5 is equivalent to the rotation and shear transformation. However the translation transformation is not implemented as it requires estimating the intercept of the asymptotes of the AA and BB clusters. However the majority of the approach is captured in the expression above.

The polynomial-based probe specific correction (PPC) approach [13] adds a probe pair index as an additional variable for the Affymetrix platform. Specifically each SNP contains 10 probe pairs, which are each calibrated by a second order polynomial mapping the three allele frequency values per probe (*f*_*AA*_, *f*_*AB*_, and *f*_*BB*_) onto 1, 0.5, 0 and interpolating between these values. Finally the 10 calibrated probe values are averaged to estimate allele frequency. Whilst the 10 probe correction is not relevant for the Sequenom data the second order polynomial mapping can be applied directly and is the following:

(5) $${} f' = \frac{\left(\,f-f_{AB}\right)\left(f-f_{BB}\right)}{\left(\,f_{AA}-f_{AB}\right)\left(\,f_{AA}-f_{BB}\right)} + \frac{1}{2} \frac{\left(\,f-f_{AA}\right)\left(\,f-f_{BB}\right)}{\left(\,f_{AB}-f_{AA}\right)\left(\,f_{AB}-f_{BB}\right)}  $$

Although the expressions for the various methods are all distinct, each expression can be decomposed into three corrections which remove distortion in the raw allele frequency response of the platform. All methods initially include a constant and linear correction to adjust the two allele frequencies corresponding to the two homozygous cases. The methods are identical at this point. Finally a method specific distortion is applied to correct the heterozygous case allele frequency. To highlight this we define an intermediate ‘homozygous corrected’ allele frequency *f* ^1^ of the form:

(6) $$  f^{1}= f + d^{1} f + d^{0}  $$

where *f* is the raw allele frequency and *d*_0_ and *d*_1_ are constants:

$$d^{0} = -\frac{f_{BB}}{f_{AA}-f_{BB}} $$

$$d^{1} = \frac{1-\left(\,f_{AA}-f_{BB}\right)}{f_{AA}-f_{BB}} $$

that enforce *f*_1_(*f*_*AA*_)=1 and *f*_1_(*f*_*BB*_)=0.

To correct the heterozygous case all methods apply a distortion correction *D* to give fully calibrated allele frequency:

(7) $$ f' = f^{1} + D\left(\,f^{1}\right).  $$

where *D* satisfies the following conditions

(8) $$\begin{array}{*{20}l} &D(0) = 0,\\ &D(1) = 0,\\ &D(f^{1}_{AB})=E,\\ &E = 0 \implies D\left(\,f^{1}\right)=0,  \end{array} $$

where $E = \frac {1}{2}-f^{1}_{\textit {AB}}$, is the error for the heterozygous case $\left (\,f^{1}_{\textit {AB}} = f^{1}\left (\,f_{\textit {AB}}\right)\right)$.

*D* is specific to each method. For piece-wise linear correction:

(9) $$\begin{array}{*{20}l} &D\left(\,f^{1}\right)=\frac{f^{1}}{f^{1}_{AB}}E & f^{1}>f^{1}_{AB}, \\ &D\left(\,f^{1}\right)=\frac{1 - f^{1}}{1-f_{AB}}E & f_{1}>=f^{1}_{AB}.  \end{array} $$

For PPC:

(10) $$  D\left(\,f^{1}\right)= E \frac{f^{1}\left(1 - f^{1}\right)}{f^{1}_{AB}\left(1 - f^{1}_{AB}\right)}.  $$

The expression for k-correction includes both *H*_*A*_ and *H*_*B*_ terms; however, these can be eliminated by solving (2) and (3) for *H*_*B*_, equating and cancelling *H*_*A*_. Furthermore after correcting homozygous cases using Eq. 6, the allele frequency for the heterozygous case is $f^{1}_{\textit {AB}} = K/(1+K)$.

The distortion term corrected by k-correction then takes the form:

(11) $$  D\left(\,f^{1}\right)=\frac{f^{1} (1-k) (1-f^{1})}{f^{1}(1-k) + k}  $$

The expression in (11) is more complicated than those in (9) and (10). If distortion in the reciprocal of allele frequency is used (11) becomes a first order distortion correction of the form in (6); however, for consistency between three methods we have expressed all in terms of allele frequency.

**Figure 1:**
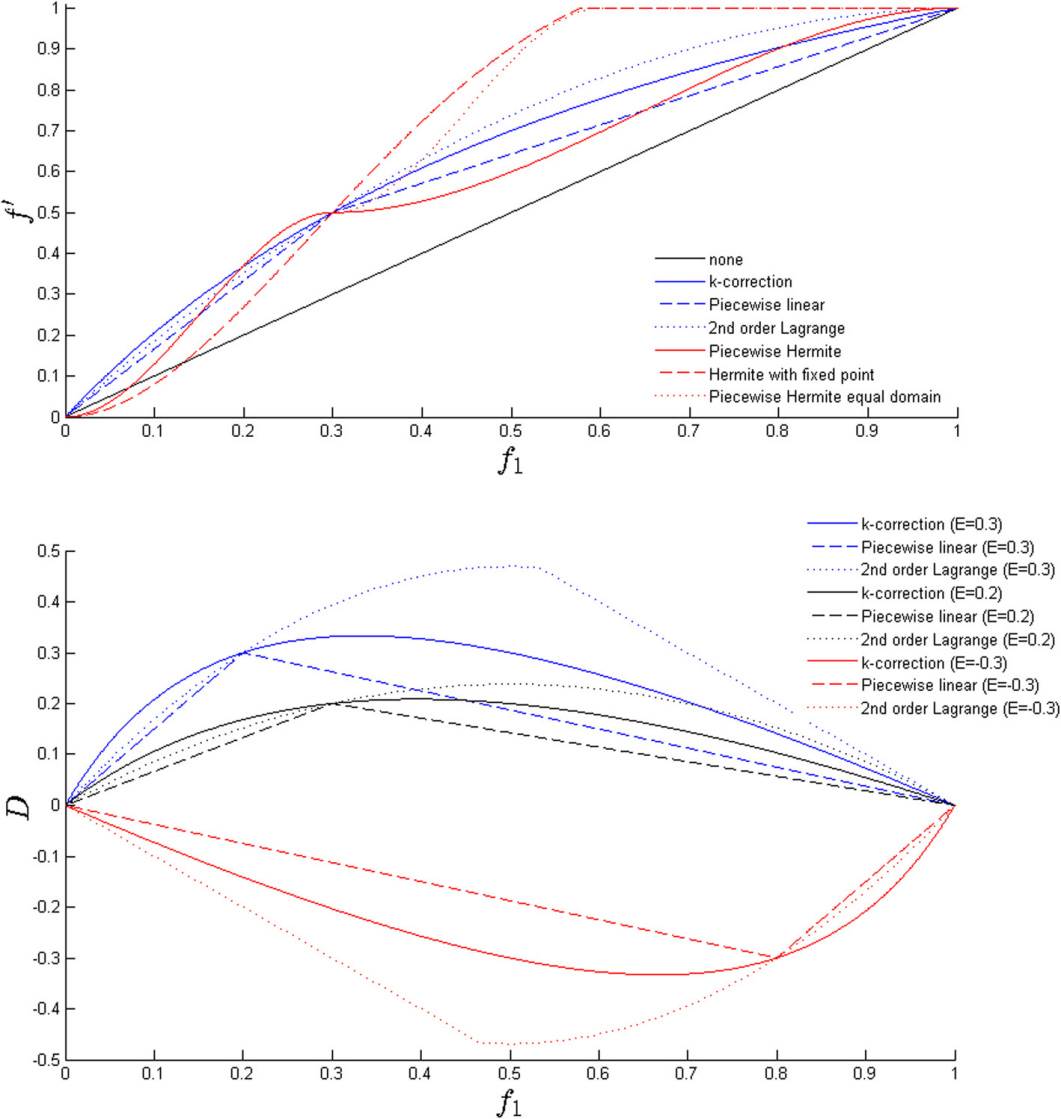
Polynomial calibration functions.**(a)** Examples of calibration functions for the heterozygous case $f^{1}_{\textit {AB}}=0.3$ **(b)** Distortion corrections for calibrations functions corresponding to *E*=0.2.

Examination of Eqs. 9, (10) and (11) show they satisfy conditions in (8). Example plots of polynomials and distortions D are given in Figure 1.

The limitations of the approaches are they all:

1. Impose assumptions on variation between 0, 0.5, 1.0,
2. address a limited set of error types.

To highlight limitation 1 we show that when testing with allele frequencies between 0, 1/2 and 1, the performance of each interpolation method varies significantly between SNPs. We then outline a machine learning technique [14] that samples across the full allele frequency range. The technique can model non-linear distortions to correct the broad range of errors that occur in the chemistry/detection processes across different genotyping platforms. Therefore they resolve the drawbacks of existing approaches. Furthermore the technique substantially reduces the measurement error. After learning the calibration the approach can be used to calibrate pooled samples measured on the same platform without further training. Finally we demonstrate the training requirements for machine learning approaches by training and testing on sets containing individuals, pools, and a combination of both.

## Method

### Experimental data

**Figure 2:**
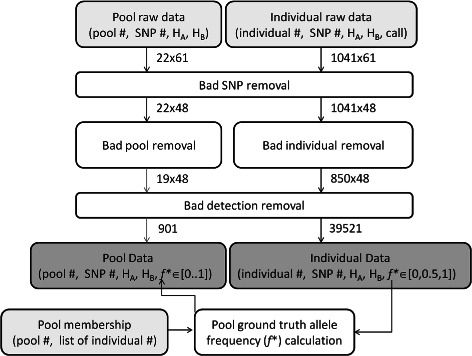
Data cleaning workflow. Workflow in cleaning input data. steps involve removing all data corresponding to bad SNPs; removing all SNP results for bad samples including both individual and pool samples. Finally low amplitude detections are removed. Size of data set shown next to arrows. Input data files shown lightly shaded. Output files dark shaded.

Experimental data from Black Tiger prawns *Penaeus monodon* was acquired from the Sequenom IPLEX platform [15]. The raw data, typical of a commercial run, generated 61 SNP (*H*_*A*_,*H*_*B*_) measurement pairs on 1041 individuals. This data was then processed for quality control Figure 2. A second experiment was conducted whereby all steps required in the genotyping process were conducted in a manner as rigorous as possible; however, due to increased cost and time of the rigorous process only a smaller set of 47 individuals were genotyped (randomly selected from the 1041 in the larger experiment). The calls from the more accurate experiment were used to rank the SNP accuracy of the larger experiment. In total 13 SNPs were identified as being inconsistent between the two experiments and were removed leaving 48 SNPs in total. The 1041 individuals were ranked in terms of number of available calls. Low quality samples (<80% calls) were removed from the data set leaving 850 samples. We also removed 1279 measurement pairs (*H*_*A*_,*H*_*B*_) values below a threshold (R<1) resulting in 39521 (*H*_*A*_,*H*_*B*_) pairs. 22 pool samples containing a minimum of 18 individuals and a maximum of 26 individuals were created from the 1041 individuals. An individual was in at most one pool sample. Three pools resulted in no data and were removed resulting in 19 pooled samples, of which 11 (*H*_*A*_,*H*_*B*_) pairs were below the minimum signal threshold and removed, leaving 901 measurement pairs. Because the pools were constructed from individuals selected from the 1041 genotyped individuals, we can calculate the ground truth allele frequencies of these pools using the 39521 individual results. A small fraction (3.4%) of the pool and SNP combinations contained individuals which did not pass quality control, and therefore were not included when calculating pool ground truth.

Finally amongst the 850 individuals 41 individuals were genotyped twice, after data quality control a total of 1621 duplicate measurements of (*H*_*A*_,*H*_*B*_) were available. These duplicate samples were used to estimate the underlying variability in the measurement process.

### Measurement error calculation

**Figure 3:**
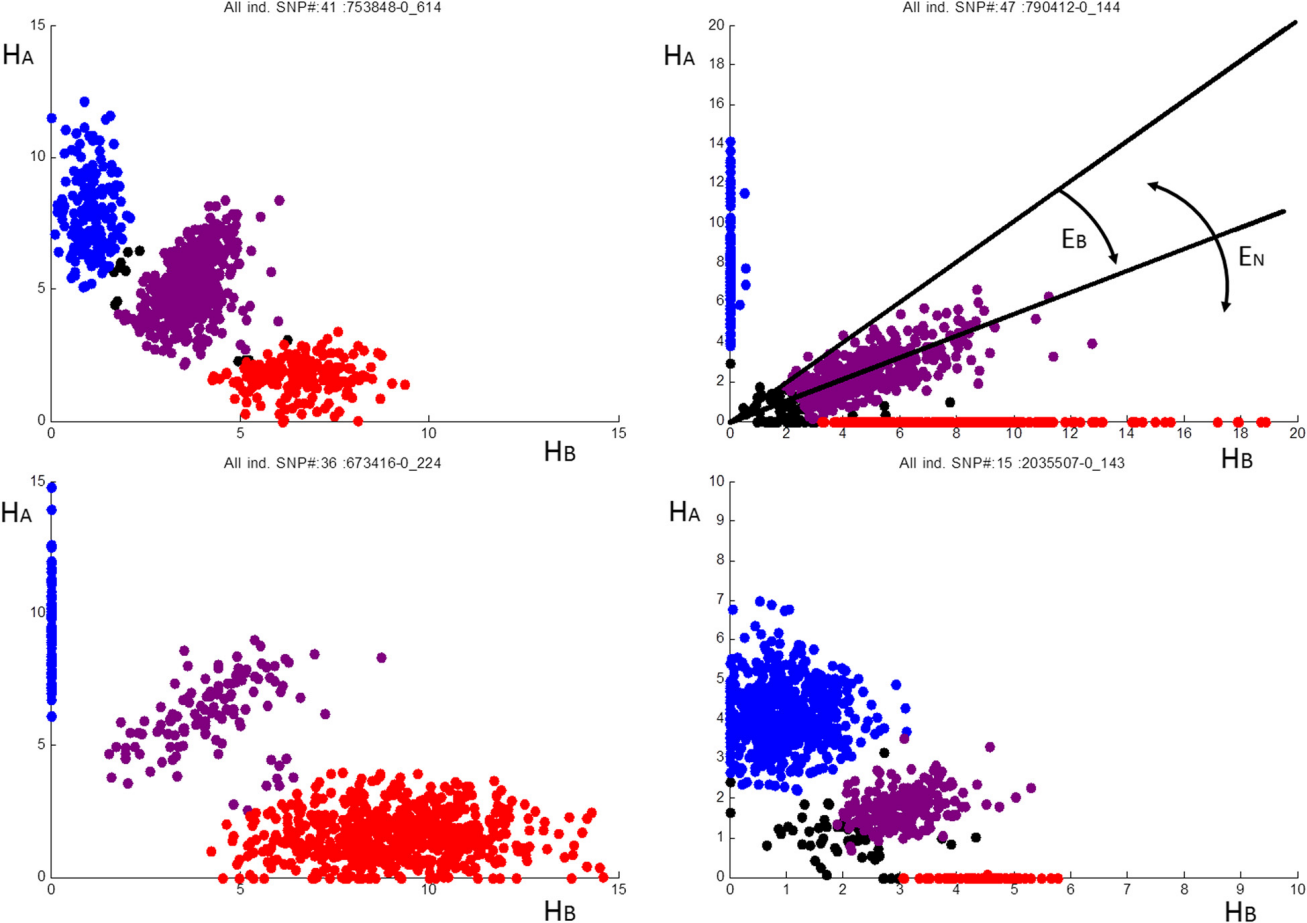
SNP coordinate pairs. (*H*_*A*_,*H*_*B*_) coordinates for the *AA* (blue), *AB* (purple), *BB* (red) cases plotted for four SNPs. Black points correspond to individual samples for which no call data is provided by platform. Pre-calibration errors *E*_*B*_(0.5) and *E*_*N*_(0.5) are shown for top right SNP.

The measurement error can be decomposed into a fixed bias term *E*_*B*_ and a random term *E*_*N*_ such that *E*=*E*_*B*_+*E*_*N*_ with corresponding MSE: ${E_{B}^{2}}+\overline {{E_{N}^{2}}}$. The bias term is the expectation of the error ($\overline {E}$) which results in an erroneous offset in the allele frequency estimate which cannot be reduced by averaging multiple estimates, the variance in measured allele frequency is $\overline {{E_{N}^{2}}}$ and both errors are functions of allele frequency: *E*_*B*_=*E*_*B*_(*f*), *E*_*N*_=*E*_*N*_(*f*). In particular *E*_*N*_(0),*E*_*N*_(1/2),*E*_*N*_(1) can be directly measured using individual samples with allele frequencies 0, 0.5 and 1 respectively. For example, Figure 3 shows the uncalibrated cluster of points corresponding to heterozygous individuals, *E*_*N*_(1/2) corresponds to the angular spread of the cluster and *E*_*B*_(1/2) to the rotation of the cluster centre from the ideal 45 degree angle. An optimal pooling strategy involves minimising the combination of *E*_*S*_,*E*_*P*_ and *E*_*N*_. Intuitively the strategy should balance the contributions from different sources, significant reduction of any single error below that of other errors has limited benefit. The optimal strategy is dependent on a combination of the expected allele frequencies and trait probabilities, and can be found based on information loss in the process of pooling [16].

To test our methods we introduce three testing regimes: *individuals*, *pools* and *combined*. The regimes evaluate the performance of the calibration methods by testing with samples that are either all individuals, all pools, or a combination of individual and pool samples. The combined set is constructed such that the error incurred on the combined set contains equal contributions from pools and individuals. Although the presented methods are developed to be applied to data sets containing pools, we include a data set comprising individuals only. The intent is not to provide results indicative of application of the methods, but to demonstrate the performance of the methods at detector values typical of homozygous and heterozygous samples. The *individual* regime highlights the contrast in performance with the existing methods developed with individual data in addition to allowing error to be decomposed into bias and variance components due to the presence of multiple calibrations at the same allele frequency.

**Figure 4:**
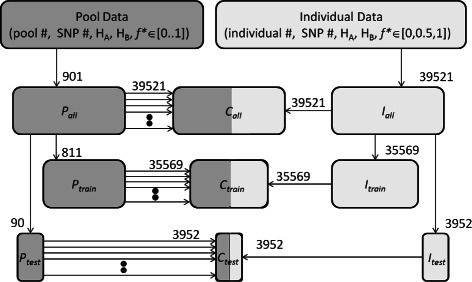
Data set generation. Workflow for generating data sets for the various training and testing regimes are shown. Numbers correspond to number of (*H*_*A*_,*H*_*B*_) pairs copied to data set. Pool samples dark shading, individual sample light shaded. Pool samples are copied multiple times to mixed data sets to ensure equal representation in mixed data sets.

The test data sets are named *I*_*all*_, *P*_*all*_ and *C*_*all*_. *I*_*all*_ contains all 850 individuals, *P*_*all*_ contains all pool data. *C*_*all*_ contains all the samples in *I*_*all*_ and *P*_*all*_; however, to ensure equal number of pooled samples, samples from *P*_*all*_ are replicated either 43 or 44 times into *C*_*all*_ until pool samples comprise an equal proportions of the data set. See Figure 4.

### Polynomial calibration

Three existing techniques were applied: linear interpolation, k-correction and 2nd order Lagrange interpolation. In addition we also implemented three variations of Hermite interpolation to explore whether alternative interpolating functions could achieve better corrections on the Sequenom platform. The techniques are equivalent to the existing methods in mapping the homozygous cases as in (6), with a distortion specific term *D* satisfying conditions in (8). Piecewise Hermite interpolation implements two Hermite polynomials over sub-domains [0,*f*_*AB*_] and [*f*_*AB*_,1] and enforces a derivative at *f*=0, *f* = *f*_*AB*_, and *f* = 1. We enforce zero derivative in our implementation. An equal domain version creates a symmetric function either side of *f*_*AB*_, finally a fixed point variation of the equal domain version enforces the derivative to be unit valued at *f*_*AB*_. To highlight the differences in calibration polynomials the functions are plotted in Figure 1 for the case of correcting an erroneous heterozygous allele frequency measurement *f*_*AB*_=0.3.

The MSE in allele frequency was calculated by calibrating under the three regimes described previously: *individuals*, *pools* and *combined*.

### Machine learning approaches

The new approach outlined in this paper utilises machine learning techniques to learn functions that correct and estimate allele frequency. Three approaches were implemented including linear regression (LR), multilayer perceptron (MLP), and support vector regression (SVR). WEKA implementations of the LR and MLP algorithms were used [17] and libSVM [18] used for the SVR. Each method learns a function that maps *f* into a calibrated allele frequency output *f* ^′^. The training data set (which includes samples of *f* and known ground truth allele frequencies *f*∗) is used to learn the mapping function. The methods find different solutions to the function due to the fact the methods impose differing constraints on the solution and optimise different objectives. LR finds the linear representation that minimises the least squares error over the training set and requires no additional parameters to define the approach. Both MLP and SVR learn non-linear mappings and require a number of parameters to define both the type of function representation, and the optimisation approach.

The MLP [19] is implemented as a cascaded series of matrix-vector multiplications. The ’vector’ input to the first matrix-vector product is the uncalibrated allele frequency value. A non-linear operation is applied to the output of each matrix-vector multiplication, the result is then multiplied by the next matrix in the series. The output of the final matrix-vector product is the calibrated allele frequency value.

Therefore the function representation is defined by parameters describing the number of matrix vector products (number of layers) and the length of the vectors (number of hidden nodes) resulting from the matrix vector products (notwithstanding the final output ‘vector’ length which is prescribed and of length one in this case). Furthermore a parameter specifying the type of non-linearity applied at each layer is required. Optimisation involves finding the values of the matrix elements (weights) that minimise an objective function. Typically a regularisation parameter is included to ensure the weights do not overfit the training data by finding an exact match, additional parameters specify the search method. Here we use a gradient based search with learning rate and momentum describing the update set. Specific values for parameters are shown in Table 1.

**Table 1:** **Parameters describing machine learning approaches**

**Multi-layer Perceptron (MLP)**			
**Parameter**	**Best**	**Range minimum**	**Range maximum**
num layers	2	1	3
nodes per layer	2	1	6
learning rate	0.11	0.01	1.0
momentum	0.15	0.01	1.0
Non-linearity	Sigmoid in all hidden layers		
**Support Vector Regression (Lib SVM nu-SVR)**			
nu	0.092	0.01	1.0
C	0.027	0.01	1.0
Kernel	Gaussian		

Support vector regression builds a function based on the training data itself. The function is represented as a sum of non-linear basis functions (called kernels) centred at each training sample. Parameters are required to describe the choice of kernel, the cost function and the optimisation approach. A common choice of basis function are Gaussians with a specified standard deviation in the input domain. The SVR cost function has no cost for small errors, this allowable error can be explicitly provided as in the *ε*-SVR, or implicitly provided via a parameter *ν* in the nu-SVR which finds a balance between regularisation and *ν*. Here the nu-SVR [20] is used with the parameters provided in Table 1. Where parameters are not explicitly stated, default parameters provided by WEKA and lib-SVM were used.

Whereas the polynomial calibration used cluster centres for determining the calibration polynomials, machine learning directly use the sample values as training data. The question arises as to what proportion of pooled data should be used versus the individual data. To examine this we introduce three training regimes *individuals*, *combined* and *pools*, which train the models using data from the respective data sources. The intent of the machine learning approaches is to provide samples away from the homozygous and heterozygous sample cases, to improve the calibration in these regions; however, we provide the *individuals* training set to allow comparison with existing methods which rely on samples from individuals only, and also provide the ability to decompose errors into variance and bias components in the resulting *f* ^′^.

An additional requirement on the data sets to ensure valid results for the machine learning approach is there is no intersection between the data used for training the models and the data used for testing the models. To achieve this the data sets are further refined. Specifically we use a cross-validation approach: the original data set containing all the data is partitioned into 10 blocks. One block is removed for testing and the remaining 9 blocks used for training. Consequently we create two pool data sets *P*_*train*_ and *P*_*test*_ which partition *P*_*all*_, and individual sets *I*_*train*_ and *I*_*test*_ which partition *I*_*all*_. Similar to the *combined* testing regime, we create data sets *C*_*train*_ and *C*_*test*_, resampling from *P*_*train*_ and *P*_*test*_ to ensure equal representation by pooled samples in the combined data sets. The process for generating the data sets is shown in Figure 4.

The data sets used in training the machine learning approach and used for calibration are dependent on the training and testing regimes and shown in Table 2.

**Table 2:** **Sets used for machine learning under different regimes in format: (training sets ; testing sets)**

	**Test regime**		
**Train regime**	***Individuals***	***Combined***	***Pools***
*individuals*	(*I*_*train*_ ; *I*_*test*_)	(*I*_*train*_ ; *I*_*test*_ + *P*_*all*_)	(*I*_*all*_ ; *P*_*all*_)
*combined*	(*I*_*train*_ + *P*_*all*_ ; *I*_*test*_)	(*C*_*train*_ ; *C*_*test*_)	(*I*_*all*_ + *P*_*train*_; *P*_*test*_)
*pools*	(*P*_*all*_ ; *I*_*all*_)	(*P*_*train*_; *P*_*test*_ + *I*_*all*_)	(*P*_*train*_; *P*_*test*_)

## Results and discussion

The pairs of duplicate measurements were used to calculate the underlying variation in the measurement process which cannot be removed by calibration. The difference in duplicated measurements *d* is a random variable with twice variance of the allele frequency measurement. Given *m* duplicate measurements the variance is $\sum d^{2} / 2m$. After data cleaning *m*=1621 duplicate samples remained. The measurement process was found to contribute a variance component of 1.91×10^−3^ to $\overline {{E_{N}^{2}}}$.

The results for standard calibration techniques are shown in Table 3. Mean square errors are averaged over SNPs and samples, by summing over all (*H*_*A*_,*H*_*B*_,*f*∗) entries in the respective testing data sets. Due to the fact that calibration polynomials map cluster centres to exact allele frequency, bias error is small and the majority of error is random *E*∼*E*_*N*_. For the case of no calibration the bias error is larger than the random error (${E_{B}^{2}}=5.56\times 10^{-3}$). Each polynomial’s ability to calibrate is highly SNP dependent. The proportion of SNPs for which each approach achieved superior results (in comparison to the other approaches) is shown in Table 4. An optimal approach might select the best calibration function on a SNP by SNP basis, such an approach would attain the results shown in the bottom row in Table 3. Table 4 clearly shows the optimal form of the calibration function differs across SNPs. For example, although the SNPs shown in Figure 3 exhibit similar cluster centres, the distribution of points in the clusters are significantly different. Existing calibration approaches operate only on the three cluster means and not the distributions. The proposed machine learning approach operates on the clusters as well. Care should be taken in interpreting Table 4, for example ‘doing nothing’ yields the most accurate results 35.4% of the time. Clearly many of the SNPs raw data is already accurate and calibration degrades the data. However, the introduced errors on these SNPs are more than compensated by error reduction across the remaining SNPs resulting in calibrated MSE errors well below that of the ‘do nothing’ case.

**Table 3:** **Allele frequency MSE (** ***10*** ^***3***^ **) obtained by calibration polynomial methods**

**Method**	***Individuals***		***Combined***	***Pools***
	*E* ^2^	$\overline {{E_{N}^{2}}}$		
None	8.83	3.27	12.32	15.80
k-correction	4.26	3.74	8.35	12.44
Piecewise linear	4.07	3.72	8.23	12.38
2nd order Lagrange	4.21	4.01	8.28	12.34
Piecewise Hermite	2.68	2.40	8.73	14.77
Piecewise Hermite equal deriv.	7.62	7.54	14.16	20.69
Piecewise Hermite equal domain	3.54	3.45	10.27	16.99
Best approach applied per SNP	2.58	2.45	7.57	11.34

**Table 4:** **Percentage of SNPs where given method obtains best performance**

**Method**	***Individuals***	***Combined***	***Pools***
None	0	14.6	35.4
k-correction	4.2	27.1	29.2
Piecewise linear	0	14.6	10.4
2nd order Lagrange	0	8.3	14.6
Piecewise Hermite	85.4	22.9	4.2
Piecewise Hermite equal deriv.	0	2.1	2.1
Piecewise Hermite equal domain	10.4	10.4	4.2

The machine learning approaches were trained and tested as described earlier. Results are shown in Table 5. Numbers in parentheses after the testing set names correspond to the worst case standard deviation of the errors over cross validation folds in the column corresponding to the test set. For the *individuals* testing regime the error was decomposed into bias and variance components, and total mean square error *E* ^2^ and variance ${E_{N}^{2}}$ are provided.

**Table 5:** **Machine learning allele frequency MSE’s (** ***10*** ^***3***^ **)**

**Method**	**Training set**	***Individuals*** ** (0.6)**		***Combined*** ** (0.7)**	***Pools*** ** (2.2)**
		***E*** ^***2***^	$\boldsymbol {\overline {{E_{N}^{2}}}}$		
	*individuals*	4.56	3.12	7.58	10.58
LR	*combined*	6.10	2.72	6.66	7.44
	*pools*	32.93	1.15	19.03	5.28
	*individuals*	2.58	2.01	8.90	15.10
MLP	*combined*	4.96	2.42	6.34	8.00
	*pools*	16.35	2.17	10.92	5.91
	*individuals*	4.22	2.68	6.78	9.29
SVM	*combined*	6.64	2.55	6.55	6.54
	*pools*	10.05	2.37	8.40	7.05

The reason for generating testing and training sets including mixtures of individuals and pools is evident in the results. Examination of just one testing set can lead to erroneous conclusions on performance. For example piecewise Hermite polynomials achieved the best results in Table 3 for minimising variance in individuals. However, this is a result of the zero derivatives enforced at 0, 0.5 and 1, which tend to compress the results towards the correct allele frequencies. The disadvantage of this is seen with the larger errors incurred when testing with pools. A similar, overfitting effect, occurs for learning models trained on *individuals* which result in flattening of the mapping in the AA, AB, BB cluster regions. The non-linear MLP and SVR methods can achieve this flattening, whereas LR cannot. Consequently MLP and SVR trained on *individuals* achieved poor results when tested on *pools* in contrast to LR.

The effect of changing the number of pools and individuals was also explored. The linear regression approach was applied to two scenarios, using *individuals* for training and testing, and using *pools* for training and testing. The results showed the existing method’s MSE was improved upon if at least 10 individuals and 8 pools were included in the respective training sets. Improvement stopped after 225 individuals were included. The available pools data set was not large enough to see performance stop improving.

In summary, whereas existing calibration approaches are trained using individual samples, machine learning approaches should not, and pooled samples are required. There is an advantage in including calibration pools when building calibration models. However care must be taken to avoid learning near the pool allele frequency values only. Models that achieved the best results (when tested on pools) were those trained only on the calibration pools and were not accurate elsewhere over the full allele frequency range. This is highlighted by the larger errors committed by all methods when trained on *pools* and tested on *individuals*. A typical experiment will involve calibration pools (with known ground truth allele frequency) and phenotype pools to be corrected. The spread of the calibration pool allele frequencies is determined by the allele frequency of the population the pool is taken, and the size of the pool. However, for phenotype specific pools being calibrated there is no guarantee a SNPs allele frequency lies within this spread, particularly if there exists a relationship between the SNP and phenotype. Therefore ideally a calibration function should be accurate over the full range of allele frequencies [0,1], or alternatively be only applied within the spread of allele frequencies on which the model was built. One alternative is to use smaller number of samples in constructing calibration pools, to increase spread. Another solution is to include a mixture of pools and individuals in the training of the algorithm such as the combined data set.

The most accurate method applied to pools, when trained with allele frequencies on the *combined* set was the SVR which achieved a MSE of 6.54 ×10^−3^ on *pools* with a variance of 2.55 ×10^−3^ on *individuals*. This is 33% larger than the duplicate values random error so although some scope still exists for improvement in reducing variation, any future substantial improvements would require investigation of the causes of variation in the platform response to duplicate samples. The best polynomial calibrator (2nd order Lagrange) achieved 12.34 ×10^−3^ MSE on *pools only* with much larger variance of 4 ×10^−3^×10^−3^ on *individuals only*. This is almost a factor of 2 in error reduction in MSE by the machine learning approach. The best approach for minimising variance on the *individuals only* set was the MLP which achieved a random squared error component 2.17 ×10^−3^ comparing with calibrated MSE of 3.75 ×10^−3^. This corresponds to an increase in increase in test statistic of 72% compared to standard calibration on the *pools only* data set. A comparison of the best performing ML approach with the existing methods is given in Table 6.

**Table 6:** **Ratio of the best machine learning approach MSE to the best existing technique MSE for each training and testing set combination**

	**Testing set**		
**Training set**	***Individuals***	***Combined***	***Pools***
*individuals*	0.63	0.82	0.75
*combined*	1.22	0.77	0.53
*pools*	2.47	1.02	0.43

## Conclusion

This is the first study of a machine learning approach to calibration of pooled SNP samples which has demonstrated the importance of training sample location on performance. The approach was tested on data generated by a Sequenom iPLEX SNP panel providing results for 61 SNPs on Tiger prawn individual and pooled samples. We showed that SNP to SNP variation is significant between the allele frequencies and different calibration polynomials are suitable for different SNPs. We introduced a machine learning technique to model each SNP separately and included data between the discrete allele frequencies of individuals by incorporating calibration pools into the model. The machine learning approach achieves significantly less error, by reducing error by a factor of 2 and improves study test statistic by 72% as a consequence of reduction in allele frequency variance.

An additional advantage of the machine learning technique is the ability to calibration functions on higher dimensional inputs. The use of additional input information can allow errors which previously created variance in *f*, to become predictable in the additional dimension. In this situation variance causing error is converted to a bias error which can be corrected by calibration with a resulting reduction in variance. Here we have limited access to auxiliary data from the experiment and using allele frequency alone has allowed comparison of the techniques with the same input data.
